# Regulation of mRNA decay in plant responses to salt and osmotic stress

**DOI:** 10.1007/s00018-016-2376-x

**Published:** 2016-09-27

**Authors:** Dorota Kawa, Christa Testerink

**Affiliations:** Plant Cell Biology, Swammerdam Institute for Life Sciences, University of Amsterdam, Postbus 94215, 1090 GE Amsterdam, The Netherlands

**Keywords:** Osmotic stress, Salinity, mRNA decay, mRNA decapping, mRNA stability, 5′→3′ exoribonucleases, SnRK2 kinases, Protein phosphorylation, Posttranscriptional regulation, P bodies

## Abstract

Plant acclimation to environmental stresses requires fast signaling to initiate changes in developmental and metabolic responses. Regulation of gene expression by transcription factors and protein kinases acting upstream are important elements of responses to salt and drought. Gene expression can be also controlled at the post-transcriptional level. Recent analyses on mutants in mRNA metabolism factors suggest their contribution to stress signaling. Here we highlight the components of mRNA decay pathways that contribute to responses to osmotic and salt stress. We hypothesize that phosphorylation state of proteins involved in mRNA decapping affect their substrate specificity.

## Emerging role of posttranscriptional level regulation in osmotic stress responses

**Fig. 1:**
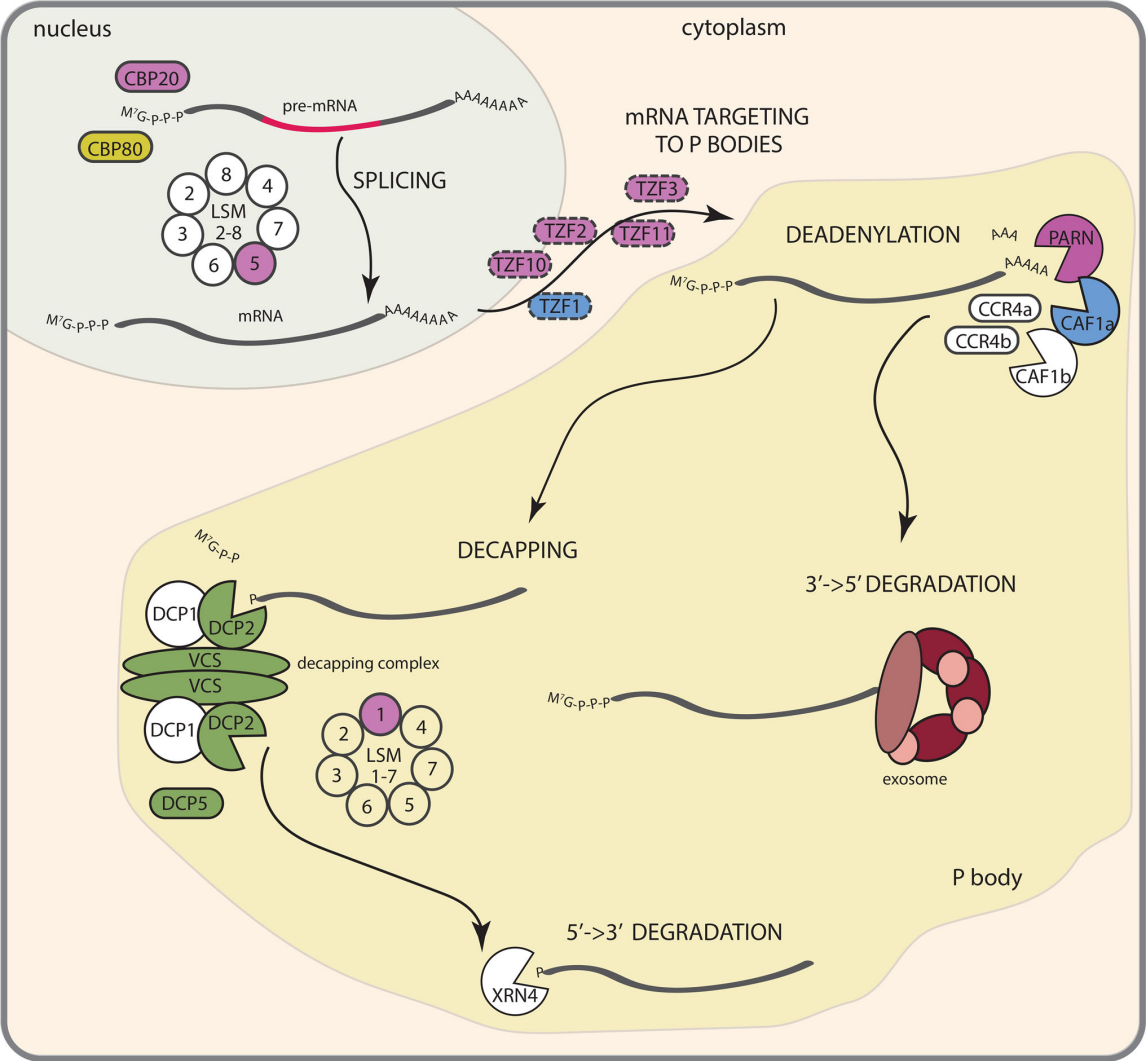
Contribution of mRNA metabolism processes to plant responses to salinity and osmotic stress. Control of splicing occurs in the nucleus and is controlled by CBP20, CBP80 and the LSM2-8 complex. Targeting of specific transcript subsets to P bodies is hypothesized to be guided by TZF proteins. Cytoplasmic mRNA decay starts with deadenylation. After shortening the poly(A) tail, transcripts can be degraded from their 3′ end via the exosome complex or undergo 5′ cap removal in a process of decapping followed by 5′→3′ decay catalyzed by XRN4. Proteins marked in *blue* and *green* are involved in responses to salt and osmotic stress, respectively, *purple color* denotes factors involved in salt, osmotic and ABA signaling, while *yellow* indicates proteins responding to osmotic stress and ABA. Proteins for which function is only hypothetical are marked with *dashed circles*

Plant survival under suboptimal conditions relies on the perception of environmental signals followed by signal transduction pathways leading to changes in gene expression to switch on protective mechanisms. Among the abiotic stresses with a deleterious effect on plant development, salinity and osmotic stress are huge constrains for agriculture worldwide [1]. The decrease in osmotic potential of the soil that occurs both in saline soils and during drought leads to a reduction in water uptake by the plant. Plant adaptations to dehydration include stomatal closure, regulation of water fluxes and biosynthesis of osmoprotectants. To cope with salinity, in addition ion homeostasis needs to be adjusted [2, 3]. Both salt stress and low water availability lead to alteration in shoot and root growth [4, 5]. These physiological changes are guided through complex signal transduction pathways including Ca^2+^, reactive oxygen species and lipid signaling, abscisic acid (ABA) formation, sucrose-non-fermenting 1-related protein kinase 2 (SnRK2) and mitogen activated protein kinase (MAPK) activation, and transcription factor regulation [2, 4, 6]. Recent research has identified the regulatory networks of the osmotic stress-induced AREB/ABFs and DREB2A transcription factors (reviewed in [7, 8]), and transcriptomic studies have identified many transcriptionally regulated candidates genes for osmotic and salt stress responses [9, 10]. However, abundance of mRNA depends not only on the control of transcription, but also on transcript degradation rate. Salt induced changes in Arabidopsis transcript abundance do not always correlate with observed changes in the proteome and most of the mRNAs for which abundance decreases on dehydration also show lower assembly with polysomes, suggesting posttranscriptional regulation of osmotic and salt stress responses [11, 12]. Besides translation, another fate of transcribed mRNAs is to be subjected to mRNA decay or quality control pathways [13]. Increasing knowledge about factors regulating mRNA turnover in plants suggests their contribution to developmental processes [14–16] as well as stress responses [17]. Altered sensitivity to osmotic stress or salinity of mRNA decay machinery mutants [17], together with the short half-life time of transcripts involved in osmotic stress responses [18], suggests mRNA decay regulation to be an additional, important level of osmotic and salt stress signaling. Moreover, SnRK2 and MAP kinases known to be involved in salt and osmotic responses seem to interfere with mRNA stability pathways [19–22]. Here, we summarize current knowledge on mRNA metabolism contribution to drought and salt stress signaling and put these results into perspective of the action of already well-known players in these pathways (Fig. 1).

## Global mRNA stability under osmotic stress

Many stress responsive genes, including ones involved in osmotic stress in human cells [23], yeast [24–27] and plants [17, 28, 29] has been shown to be regulated by their mRNA stability. In yeast, the dynamics of global transcriptional rate after osmotic stress does not always match the changes in dynamics of mRNA abundance, which can be explained by global transcript destabilization [27]. Stability of mRNA was shown to depend on stress severity. Mild stress in general caused mRNA decay, while most osmotic stress-responsive mRNAs were stabilized, suggesting that regulation of mRNA turnover can affect specific subsets of transcripts. Severe osmotic shock resulted in global mRNA retention in processing bodies (P bodies), cytoplasmic foci where mRNA can be degraded or withheld from translation [27]. Decay of osmotic stress-induced mRNAs in yeast has been proposed to be essential in the recovery phase, to return to the base level of these mRNAs [30].

In Arabidopsis, mRNAs coding for proteins involved in responses to osmotic stress have been found among rapidly degraded transcripts [18]. Around 40 % of mRNAs encoding sodium transporters were found in their uncapped version, implying that 5′→3′ mRNA decay can also regulate responses to salt [31]. Global analysis of the transcriptome and degradome of *Setaria italica* revealed that also in this monocot species 5′→3′ mRNA degradation is one of the mechanisms of responses to drought [32]. While control of mRNA stability in response to osmotic stress is receiving increasing attention, our knowledge of the mechanisms behind observed changes is still fragmentary.

## mRNA decay pathways in Arabidopsis

Control of mRNA stability is an essential component of gene expression regulation. The rate of transcript degradation correlates with encoded function, and abundance of the transcript, number of introns and conserved sequences in the 5′ and 3′ UTR [18]. Arabidopsis mRNAs coding for transcription factors and protein kinases as well as transcripts targeted by microRNAs, and low abundance transcripts are in general unstable. Presence of at least one intron increases mRNA stability, and multiple stabilizing motifs within the UTRs’ sequences have been identified [18]. Most transcripts appear in complex with proteins involved in translation initiation or mRNA decay and are constantly shifted between these two processes. Factors that activate the first steps of mRNA degradation in general repress translation and vice versa [33, 34]. The mRNA decay machinery can target defective transcripts with premature stop codons or long 3′UTR in nonsense-mediated decay (NMD), mRNA molecules without a stop codon in the process of non-stop decay (NSD), as well as transcripts that did not disassociate from ribosomes, by a mechanism known as non-go decay (NGD) (reviewed in [35, 36]). Besides degradation of abnormal transcripts, the mRNA decay machinery can also target mRNAs with a correct structure, serving as a regulatory step in gene expression [37]. The same enzymes are involved in mRNA degradation in specialized and general pathways (Fig. 1), but the recognition of substrates follows different mechanisms [37].

### First step—deadenylation

In all eukaryotic organisms mRNA degradation starts by shortening the 3′ poly(A) tail [35]. This critical and rate-limiting step is guided by the deadenylases PARN, PAN and the CCR4/CAF1/NOT complex [38]. The poly(A) ribonuclease AtPARN catalyses deadenylation of a subset of embryonic transcripts in Arabidopsis, and lack of functional AtPARN results in embryo lethality [39, 40]. Expression of AtPARN is induced by ABA, osmotic and salt stress, while several AtPARN substrates were found to be degraded in both stress and control conditions [41]. Two Arabidopsis homologs of CARBON CATABOLYTE REPRESSOR4 (AtCCR4a and AtCCR4b) were shown to be involved in starch and sucrose metabolism [42, 43]. Eleven Arabidopsis CCR4-ASSOCIATED FACTOR1 (CAF1) genes have been found, among which AtCAF1a and AtCAF1b, which are associated with responses to multiple environmental stimuli, in most cases by non-redundantly targeting different mRNA subsets [44]. A mutant in AtCAF1a was able to germinate in the presence of salt and one-third of the transcripts upregulated in this mutant were salt-inducible, including one aldehyde dehydrogenase, ALDH7B4, which upon overexpression led to the same phenotype as the *caf1a* mutant [44, 45]. Important to note is that the paralogous deadenylase AtCAF1b did not share a function in responses to salt, suggesting that similar enzymes with the same function can target different transcripts and thereby regulate other stress responses [44]. Neither the poly(A) nuclease PAN, that functions in deadenylation in humans and yeast, or other components of the CCR4/CAF1/NOT complex have been identified in Arabidopsis [37, 38].

### 3′→5′ degradation

Transcripts deprived of their poly(A) tail can be targeted to 3′ end decay catalyzed by the exosome, a multisubunit complex involved in cytoplasmic mRNA degradation, and maturation of rRNA, snRNA and snoRNA in the nucleus [46]. The exosome is comprised of nine core subunits and associated cofactors including RNA-binding proteins and RNA helicases [37]. On the contrary to yeast and humans, the plant exosome core complex is self-activated through the phosphorolytic function of AtRRP41 (*Arabidopsis thaliana* homolog of *Saccharomyces cerevisiae* exosome subunit RRP41p) [47]. Mutations in exosome proteins result in early growth, female gametogenesis and embryogenesis defects [47–49], but so far no evidence has been reported for a role of the exosome machinery in osmotic stress signaling.

### 5′→3′ degradation starts with decapping

Deadenylated transcripts can be also directed to 5′→3′ decay, where 5′ cap removal is the first step [13]. In Arabidopsis, this process is catalyzed by DECAPPING 2 (DCP2), acting in a complex with its activator DECAPPING 1 (DCP1), which does not have decapping activity itself. The DCP2-DCP1 interaction is crucial for 5′ cap removal and depends on VARICOSE (VCS) as a scaffolding protein [15]. Impaired function of decapping complex components results in severe developmental phenotypes, including cotyledon deformation or improper vein pattering, and lethality of homozygous knock-out (KO) mutants [16, 50]. Decapping and further degradation take place in processing bodies (P bodies), dynamic cytoplasmic foci concentrating mRNA decay enzymes, which depend on the supply of mRNA for their formation [15, 16, 51]. Besides the decapping machinery, another protein complex, consisting of LSM1-7 proteins, has been shown to serve as a decapping activator. Eight highly conserved SM-Like (LSM) proteins have been identified in Arabidopsis, which via specific interactions with each other, form two separate heptameric complexes: cytoplasmic LSM1–7 and nuclear LSM2–8, the latter functioning in splicing events [52, 53]. LSM 2, LSM3, LSM4, LSM5, LSM6 and LSM7 are present both in cytoplasm and nucleus, while LSM1 and LSM8 localization is restricted to cytoplasm and nucleus, respectively [52]. Two duplicated functionally redundant genes, *LSM1A* and *LSM1B*, encode for the LSM1 protein [52, 53]. In an *lsm1a lsm1b* double mutant decapping and decay rates of transcripts was decreased [28, 52]. Another protein carrying an LSM domain, DCP5, was shown to promote decapping without affecting DCP2 activity [54]. The formation of P bodies was altered in both *dcp5* and *lsm1a lsm1b* mutants, suggesting that DCP5 and the LSM1–7 complex may indirectly activate decapping via P body assembly [52, 54]. Two other LSM proteins, LSM4 and LSM5/SUPERSENSITIVE TO ABA AND DROUGHT 1 (SAD1), have been described only for their role in splicing processes so far [53, 55, 56].

While proper functioning of the decapping complex is required for basal plant development, an increasing amount of evidence suggests it may also be involved in stress responses [22, 28, 29, 52]. Stability of osmotic and salt-responsive transcripts appears to be regulated via DCP1 and its interaction with other proteins. Within 15 min upon dehydration, MAP kinase 6 (MPK6) is activated and phosphorylates DCP1. Phosphorylation of DCP1 then triggers DCP1 dimerization and association with DCP2 and DCP5. Increased assembly and activity of the decapping complex stabilizes mRNAs encoding for signal transduction components, transcription factors and nucleic acid binding factors and induces decay of transcripts involved in lipid metabolism and nutrient balance [22]. Among the stabilized transcription factors, DREB2b is also induced by osmotic stress and high salinity [57], indicating that dehydration not only triggers expression of DREB2b transcript, but also protects it from the decay. DCP1 has also been shown to physically interact with the BEACH (*beige* and Chedial Higashi) domain protein SPIRRIG (SPI) [29]. Arabidopsis SPI, known previously to participate in maintenance of membrane integrity, was recruited to P bodies upon interaction with DCP1 [29, 58]. A *spi* mutant showed less P body formation upon salt stress, and was hypersensitive to salt [29]. SPI triggered the recruitment of a subset of salt-responsive mRNAs and ribonucleoprotein (RNP) assembly critical for P body formation. Together this suggests that under stress conditions activity of DCP1 can be modulated by interaction with MPK6 and SPI.

LSM1, one of the components of the decapping activator complex LSM1–7, was also found to guide responses to salt and drought stress. The *lsm1a lsm1b* mutant has altered levels of both hormonal signaling and stress defense transcripts [53]. Interestingly, *lsm1a lsm1b* plants showed a higher survival after drought in soil experiments and higher fresh weight and number of lateral roots on osmotic stress applied in agar media [28]. On the other hand, in the presence of salt, overall growth of *lsm1a lsm1b* seedlings was severely inhibited, suggesting their hypersensitivity to salt. Transcriptome analysis revealed that in the *lsm1a lsm1b* mutant exposed to osmotic stress, transcripts encoding positive factors for drought tolerance were stabilized (e.g. *ABR1, ANAC019, ERF53*), while salt stress led to stabilization of mRNAs of negative regulators of salinity tolerance (e.g. *ANAC092, AHK5, ATGSTU17*). One of the mRNAs stabilized specifically on salt was the ABA biosynthesis gene *NCED3,* indicating involvement of the decapping machinery in ABA biosynthesis in response to salinity. LSM1 is also crucial for formation of P bodies in osmotic or salt stress conditions. By guiding its target mRNAs to P bodies, activating their decapping and thus exposing them to degradation, LSM1 regulates sensitivity to osmotic stress and salinity [28]. Via the same processes, LSM1 regulates not only responses to drought and salinity, but also to cold stress [28]. Different stress signaling pathways target different mRNAs, yet the mechanism of this selectivity remains unknown.

### 5′→3′ cleavage

The naked mRNA molecule is an easy target for 5′→3′ exoribonucleases. While three of these have been identified in Arabidopsis, only XRN4 localizes to the cytoplasm and can target decapped mRNAs [59]. Surprisingly only a small subset of transcripts has an altered decay rate in the *xrn4* mutant and none of the previously identified highly unstable mRNAs were targets of XRN4, suggesting existence of other plant 5′→3′ exoribonucleases [18, 60]. Substrate selectivity of XRN4 was linked to the presence of hexamer motifs in the 5′ end of its targets [61]. Decapped transcripts can be also targets of RNA-dependent RNA polymerases and in this way induce gene silencing [62]. A subset of XRN4 substrates appears to be derived from miRNA cleavage, suggesting the existence of two mechanisms of XRN4 action: via general cytoplasmic 5′→3′ decay and by decay of miRNA degradation products [60]. On the contrary to mutants in decapping machinery components, *xrn4* mutants do not have any developmental alterations, but are insensitive to ethylene, show higher tolerance to heat stress and exhibit decreased sensitivity to auxin with respect to lateral root formation [63–65]. In *xrn4* mutants, levels of *EBF1* and *EBF2* mRNA, encoding F-box proteins that participate in degradation of the major transcriptional regulator of ethylene signaling EIN3, was decreased. *EBF* RNA decay rates were not altered, suggesting they are indirect targets [63, 66]. In addition, abundance of many other transcripts was decreased in the *xrn4* mutant, suggesting that XRN4 can affect transcripts level not only via its exoribonucleic activity, but also indirectly [61].

Through direct degradation of mRNA of heat shock factor A2 (*HSFA2*), XRN4 is involved in suppression of heat stress responses after return to normal temperature [64]. While the *xrn4* mutant was more tolerant to short and severe heat stress, a very low level of thermotolerance was observed to moderately high temperatures, possibly caused by separate subsets of mRNAs being targeted at different stress intensities [64, 67]. Interestingly, upon heat stress XRN4 interacts with LARP1, a homolog of human RNA-binding protein, and in this complex XRN4 is directed to polysomes [67, 68]. A proportion of the transcripts rapidly degraded upon heat stress is subjected to XRN4-dependent 5′→3′ decay while still being associated to elongating polysomes, supporting the concept of co-translational 5′→3′ decay, suggested previously to occur in yeast and Arabidopsis [67, 69, 70]. Co-translational mRNA decay was shown to be involved in salt stress signaling in yeast, and this function might be conserved in plant responses to salinity [71, 72].

## Role of nuclear mRNA metabolism regulators in salt and osmotic stress signaling

In parallel to regulators of cytoplasmic RNA metabolism, nuclear pre-mRNA processing enzymes also appear to be involved in responses to osmotic stress. Hypersensitivity to salinity of *sad1/lsm5* and *lsm4*, mutants in the nuclear LSM2–8 complex for intron removal, suggests involvement of the splicing machinery in salt stress responses [55, 56, 73]. SAD1 is essential for accurate splice site recognition and splicing efficiency. Overexpression of SAD1/LSM5 led to an increase in properly spliced transcripts of *CIPK3*, *ABF3*, and *DREB2A*, and improved salt tolerance. Aberrant splicing events of transcripts involved in salinity responses were found in *sad1/lsm5* plants exposed to salt stress [56]. A *sad1/lsm4* point mutation did not affect global gene expression, but expression of many transcripts of ABA biosynthesis and signaling pathway factors decreased [73]. Two nuclear cap-binding proteins CBP80 and CBP20 have been shown to regulate alternative splicing of transcripts coding for the auxin response factors ARF10 and ARF17, as well as for proline and sugar metabolism factors [74]. The *cbp80* and *cbp20* mutants both had higher sensitivity to salt and ABA and c*bp20* showed decreased stomatal conductance and sensitivity to water withdrawal [74, 75]. *Cbp80/abh1* mutants also had decreased levels of *PP2C* phosphatase transcripts involved in ABA signal transduction, which could explain its ABA hypersensitivity [76]. Abscisic acid (ABA) is a phytohormone with a crucial role in salt and drought signaling [77]. Both ABA biosynthesis and its signaling pathways seem to be regulated at the level of pre-mRNA splicing (also reviewed in [78]). Another nuclear protein, the mRNA capping enzyme family protein At5G28210, was mapped recently as a putative gene involved in root growth in the presence of a combination of salt and phosphate starvation [79].

## Small RNA-mediated regulation of mRNA decay

Another level of regulation of gene expression in plants is RNA interference (RNAi) in which a micro RNA (miRNA), through the RNA-induced silencing complex (RISC) can guide the cleavage of a target mRNA [80]. Products of miRNA cleavage are then degraded by XRN4 [60]. On the other hand, XRN4 acts antagonistically with RNAi processes because endonucleolytic cleavage of uncapped mRNA removes template for RNA-dependent RNA polymerases [62]. Accumulation of these transcripts in an *xrn4* mutant leads to excessive gene silencing [62]. Nuclear 5′→3′ exoribonucleases XRN2 an XRN3 suppress posttranscriptional gene silencing by degradation of the loops that are excised from a pri-miRNA during its maturation [81]. Moreover, numerous miRNAs are involved in responses to osmotic stress or salinity [82, 83] and multiple proteins participating in miRNA biogenesis are also implicated in responses to salt, osmotic stress or ABA [84].

## TZF proteins

Another group of proteins putatively involved in regulation of salt and osmotic stress responses at the posttranscriptional level are tandem CCCH zinc finger proteins (TZFs) [85, 86]. Mammalian TZFs are able to bind to ARE elements, AU-rich sequences at the 3′ end of mRNA molecules, and this interaction recruits enzymes involved in deadenylation, decapping and exonucleolitic cleavage [87, 88]. Evidence increases that plant TZF proteins may be also involved in mRNA turnover control. Arabidopsis TZF1 and TZF9 and rice OsTZF1 possess RNA binding capacity [89, 90], while TZF2 and TZF3 exhibit RNAse activity [91]. So far the contribution of plant TZFs to mRNA decay has been shown only for TZF1, which can induce degradation of ARE-containing transcripts [92].

Expression of Arabidopsis TZF1 is triggered by salt only, while TZF2, TZF3, TZF10 and TZF11 are induced by salinity, osmotic stress and ABA [91, 93]. Overexpression of TZF10 or TZF11 led to increased salt tolerance and decreased expression of RD29A and KIN1 [93]. Overexpression lines of TZF2 and TZF3 showed higher drought tolerance and reduced water loss than wild type plants [91]. Moreover, induction of TZF3 resulted in decreased expression of ABI2, while a *tzf3* knock-out mutant was hypersensitive to both salt stress and ABA [94]. Rice OsTZF1 relocalised to P bodies upon salt stress or ABA treatment and its overexpression conferred faster recovery from salinity or drought [89]. Osmotic stress-induced cotton GhZFP1 is involved in adaptation to salinity and its overexpression led to high potassium/sodium ratio. Similar to mammalian TZFs, plant TZFs can shuttle between nucleus and cytoplasm and localize to P bodies and stress granules [89, 90, 95, 96]. Importantly, altered expression of TZFs affects usually a subset of transcripts [91, 93, 94]. Specificity of mRNA metabolism processes that occur in P bodies may rely on TZF proteins binding to cis-elements in mRNA [17]. Interestingly, Y2H analysis revealed that TZF5 interacts with MARD1 and RD21A, proteins involved in ABA and dehydration responses, but not with any of the components of mRNA decay machinery [96]. This may suggest that additional interactions with stress-induced proteins may be also required for osmotic and salt regulation at the level of mRNA metabolism.

## Emerging role of protein kinases in mRNA stability regulation

**Fig. 2:**
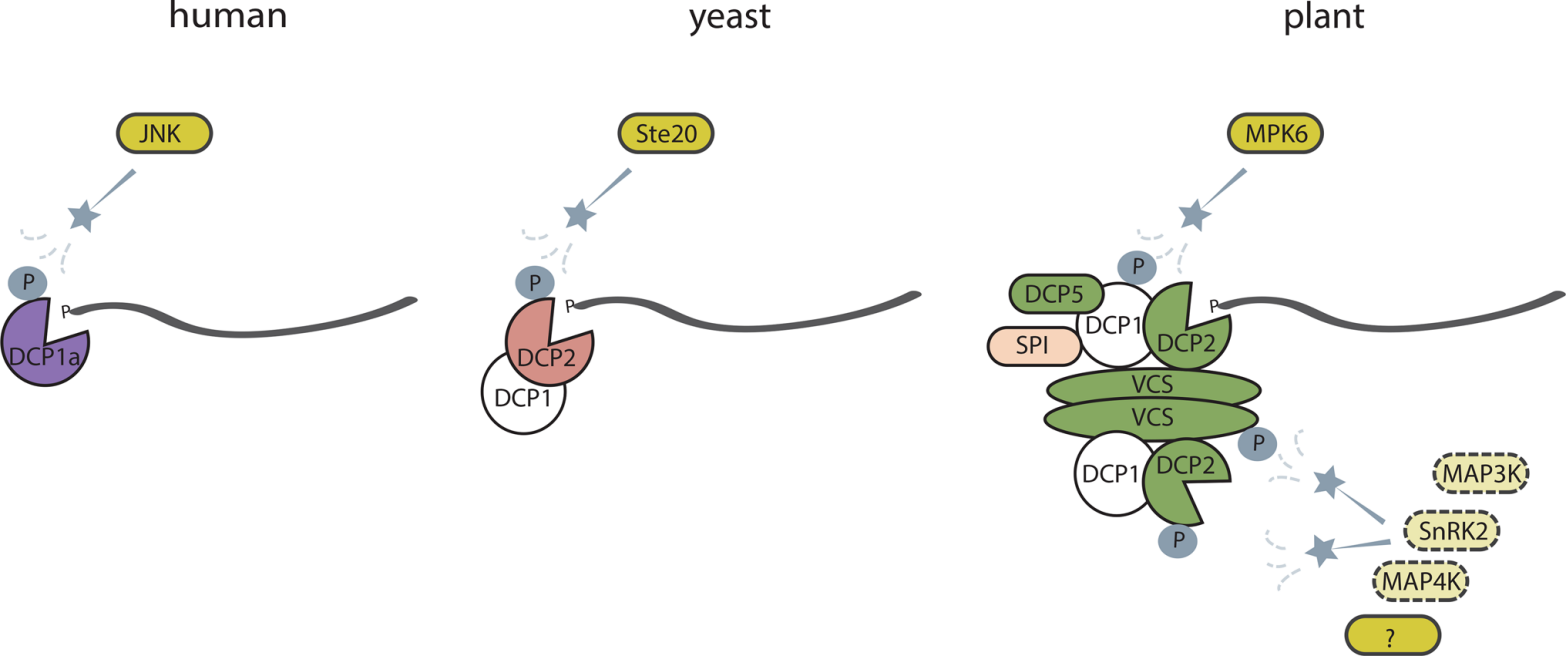
Involvement of protein kinases in regulation of mRNA decapping processes. In human cells protein kinase JNK phosphorylates DCP1a and through this regulates P body formation. In yeast, upon glucose deprivation Ste20 phosphorylates DCP2 to stabilize its interaction with DCP1. In Arabidopsis MPK6 is activated by drought and phosphorylates DCP1, thus inducing DCP1 interactions with DCP2 and DCP5. Upon salt stress, SPI protein binds to DCP1 and facilitate its recruitment to P bodies. Phosphorylation of DCP2 and VCS is triggered by osmotic stress, but kinases functioning upstream remain still unknown. Phosphoproteomic profiling studies may suggest involvement of SnRK2 or MAP kinases in this process

The aforementioned factors involved at different stages of mRNA stability can guide responses to multiple stresses by selective targeting of subsets of transcripts [22, 28, 29]. The mechanism of this stress-dependent control of differential gene expression remains unclear, but increasing recent evidence suggests that decapping factors are phosphorylated upon stress exposure. In response to osmotic stress, human DCP1a is phosphorylated by c-Jun N-terminal kinase (JNK) and targeted to P bodies [97]. In glucose-deprived yeast, the protein kinase Ste20 phosphorylates DCP2 and stabilizes the DCP1–DCP2 interaction [98]. Yeast mRNA stability upon exposure to osmotic stress is dependent on the MAP kinase Hog1, via an unknown mechanism [27] (Fig. 2).

In Arabidopsis, MPK6 is involved in phosphorylation of DCP1 in response to drought stress, while upon flagellin treatment MAP kinase 4 (MPK4) phosphorylates PAT1, another activator of decapping [22, 99]. The MPK phosphorylation motif was also found in the decapping activator LSM1, which could explain its stress-dependent substrate specificity [28]. A phosphoproteomics study revealed that DCP2 phosphorylation increases in response to osmotic stress, but the kinase catalyzing this process still remains to be identified [20] (Fig. 2).

Another member of the decapping complex, VCS, was also differentially phosphorylated upon osmotic and salt stress, but not after ABA treatment [20, 21]. The phosphorylated version of VCS was downregulated in a triple KO mutant of ABA-dependent protein kinases, the *snrk2.2/2.3/2.6* mutant, while VCS phosphorylation was lower upon ABA treatment, suggesting it could be an indirect target of SnRK2.2/SnRK2.3/SnRK2.6 [21]. Phosphorylation of multiple individual Ser residues of VCS was either enhanced or inhibited by mannitol treatment, reflecting the complicated nature of this regulation [20]. Together, this evidence suggests that differential phosphorylation of decapping factors upon osmotic or salt stress could enable them to target only a subset of transcripts. Since cytoplasmic 5′→3′ mRNA decay, starting with 5′ cap removal, occurs also in control conditions it is plausible that under osmotic stress conditions decapping activity and substrate selectivity is regulated in a different way. Evidence is accumulating for a role of post-translational modifications, including phosphorylation, in regulation of this process.

Recent phosphoproteomics studies [20, 21] have revealed upregulation of phosphorylated forms of SnRK2.1/4/5/6/10, MAP3K Raf18, MAP4Kα1 and DCP2 within 5 min after osmotic stress treatment. Nine out of ten Arabidopsis SnRK2 protein kinases are rapidly activated by osmotic stress, while activity of SnRK2.2, SnRK2.3 and SnRK2.6 is also regulated by ABA [100]. SnRK2.6 has been shown to phosphorylate the anion channel SLAC1 [101, 102], potassium channel KAT1 [103], AtrbohF NADPH oxidase [104], transcription factor ABA-RESPONSIVE ELEMENT BINDING PROTEIN 3 (AREB3) [19] and aquaporin PLASMA MEMBRANE INTRINSIC PROTEIN (PIP2;1) [105]. All SnRK2 subclass 2 and 3 can phosphorylate the transcription factor AREB1 [106]. Contrary to ABA-dependent SnRK2s, targets of SnRK2 subclass 1 protein kinases,remain to be identified. Whether these kinases or MAP3Ks/MAP4Ks can directly or indirectly target DCP2 remains unknown (Fig. 2).

It is unknown whether activity of plant DCP2 depends on its phosphorylation status, or is possibly triggered by phosphorylation of DCP1 or VCS, and finally whether phosphorylation of DCP1, DCP2 and VCS influences their relocalisation to P bodies or assembly of decapping complex. To address these questions, protein kinases targeting these proteins need to be identified. For SnRK2.6, no subcellular localization data are available, but the subclass 1 SnRK2.4 and a Medicago stress induced MAP2K (SIMKK) have been shown to localize to punctate structures after exposure of roots to salt [107, 108]. Yet, it remains to be established whether these would be P bodies or in case of SnRK2.4, would reflect lipid binding capacity [109]. Finally, it should be noted that phosphorylated versions of DCP1 and VCS have also been detected in control conditions, so any putative regulation by protein kinases in response to osmotic stress may have a quantitative character [20, 22].

Several proteins involved in pre-mRNA processing and splicing have been found as putative targets of ABA-dependent SnRK2 kinases [19, 21, 110], implying that proteins involved in nuclear mRNA metabolism can be also regulated by phosphorylation. Altered ABA sensitivity of *sad1/lsm5*, *lsm4*, *cbp20*, *cbp80/abh1*, and *ahg2*-*1* mutants suggests they function via adjustment of hormonal signaling pathways [41, 55, 73, 74, 76].

The mRNA decapping step takes place in P bodies, meaning that salt- and osmotic-dependent mRNA degradation relies on proper assembly of RNP particles and recruitment of all their components to P bodies. So far it is not known how mRNAs targeted for degradation relocate to P bodies and whether the process would involve interactions with RNA-binding proteins, decapping factors or other stress-induced proteins. The salt responsive protein SPI does not possess RNA-binding capacity but seems to regulate uptake of mRNA into P bodies and RNP formation via an unknown mechanism [29]. The LSM1 protein physically interacts not only with members of the LSM1–7 complex, but was also co-purified with some stress-responsive proteins without RNA-binding properties [53]. It would be of interest to investigate whether osmotic- and salt stress can induce interaction of decapping factors with SnRK2 kinases, MAP4Kα1, MAP3K Raf18, MPK6 or SPI and how this would modify their function.

Another possibility is that substrate selectivity of the decapping machinery depends on the abundance of targeted mRNAs, as was shown for some of the transcripts for which degradation is triggered by LSM1 [28]. Finally, specificity could also depend on the mobility of selectively targeted mRNAs. Regulation of mRNA transport from nucleus to cytoplasm is relatively well known, but the mechanism of transcript mobility in the cytoplasm and recruitment to P bodies still remains unknown [111]. RNA binding capacity of TZFs that shuttle between nucleus, cytoplasm and P bodies together with their role in tolerance to osmotic and salt stress [85] suggests that their interaction with a specific subset of transcripts could be a possible mechanism to regulate their loading to P bodies [17, 89, 90, 93, 94].

## Conclusions

Several factors controlling mRNA metabolism are involved in osmotic or salt stress responses of plants. Most of these are involved in mRNA decapping and as a consequence in 5′→3′decay of transcripts. The contribution of other factors may be indirect, for example CAF1a could function by promoting deadenylation, inducing decapping in a similar way as in yeast [112]. Interestingly, most of the factors discussed here are involved in regulation of multiple stresses, but target only a condition-specific subset of transcripts [17, 28, 41, 63]. Unraveling the mechanism of this selectivity would be of high importance. Here, we propose posttranslational modification of mRNA decay factors as a possible mechanism of substrate selectivity. Multiple phosphorylation sites have been identified in several mRNA metabolism regulators, which appear to be phosphorylated in response to osmotic and salt stress in a quantitative.way. Further characterization of osmotic- and salt-induced targets of 5′→3′decay pathways is also necessary. We propose here that changes in gene expression upon osmotic and salt stress should be studied not only at the transcriptome level, but should be extended to the posttranscriptional level.
